# Coronary artery calcium is associated with cortical thinning in cognitively normal individuals

**DOI:** 10.1038/srep34722

**Published:** 2016-10-03

**Authors:** Jin San Lee, Danbee Kang, Young Kyoung Jang, Hee Jin Kim, Duk L. Na, Hee Young Shin, Mira Kang, Jin-Ju Yang, Jong-Min Lee, Juyoun Lee, Yeo Jin Kim, Key-Chung Park, Eliseo Guallar, Sang Won Seo, Juhee Cho

**Affiliations:** 1 Department of Neurology, Samsung Medical Center, Sungkyunkwan University School of Medicine, Seoul 06351, Korea; 2 Neuroscience Center, Samsung Medical Center 06351, Seoul, Korea; 3 Center for Clinical Epidemiology, Samsung Medical Center 06351, Seoul, Korea; 4 Department of Health Sciences and Technology, SAIHST, Sungkyunkwan University, Seoul 06351, Korea; 5 Health Promotion Center, Samsung Medical Center 06351, Seoul, Korea; 6 Department of Biomedical Engineering, Hanyang University, Seoul, Korea; 7 Department of Neurology, Chungnam National University Hospital, Daejeon, Korea; 8 Department of Neurology, Chuncheon Sacred Heart Hospital, Hallym University College of Medicine, Chuncheon, Korea; 9 Department of Neurology, Kyung Hee University School of Medicine, Seoul, Korea; 10 Department of Epidemiology, Epidemiology and Clinical Research, Johns Hopkins Medical Institutions, Baltimore, USA; 11 Department of Medicine, Epidemiology and Clinical Research, Johns Hopkins Medical Institutions, Baltimore, USA; 12 Welch Center for Prevention, Epidemiology and Clinical Research, Johns Hopkins Medical Institutions, Baltimore, USA; 13 Clinical Research Design and Evaluation, SAIHST, Sungkyunkwan University, Seoul 06351, Korea

## Abstract

To evaluate the association between coronary artery calcium (CAC) and cortical thickness in a large sample of cognitively normal individuals, with special emphasis in determining if the association thickness has regional brain specificity and if it is mediated by white matter hyperintensities (WMH). A total of 512 participants were included in this study. CAC scores were assessed by multi-detector computed tomography. Cortical thickness was measured using a surface-based method. Linear mixed models were used to assess the association between CAC scores and cortical thickness. In fully adjusted models, increased CAC scores were associated with cortical thinning across several brain regions, which generally overlapped with the distribution of default mode network. The association between CAC scores and cortical thickness was significantly stronger in participants with moderate or severe WMH compared to those with none or mild WMH, even though CAC scores were not associated with WMH. In cognitively normal adults, CAC was associated with cortical thinning in areas related to cognitive function. This association was evident after adjusting for multiple coronary artery disease risk factors and for WMH, suggesting that CAC may be more closely related to Alzheimer’s Disease-type disease rather than to cerebral small vessel disease.

The pathologic mechanism underlying coronary artery disease (CAD) is atherosclerosis^1^, a generalized inflammatory disease that affects all major vascular beds, including the cerebral arteries. CAD has been proposed as a major risk factor for cognitive decline and dementia^2^,^3^,^4^,^5^.

Coronary artery calcium (CAC) detection with noninvasive computed tomography (CT) effectively identifies subclinical atherosclerosis of the coronary arteries^6^ and improves risk prediction in asymptomatic subjects at intermediate risk^7^. Few neuroimaging studies have addressed the association between CAC and brain structure^2^,^3^,^4^. These studies have suggested that CAC is not only an indicator of cerebral atherosclerosis, but is also associated with white matter hyperintensities (WMH) and with brain atrophy. Two important questions, however, remain unanswered. First, it is unclear if CAC is associated with diffuse brain atrophy or if there is regional specificity. Second, the mechanisms linking CAC to brain atrophy are unknown. Since WMH is associated with brain atrophy^8^, it is reasonable to expect that the association between CAC and brain atrophy may be mediated by WMH. Alternatively, CAC may be associated with brain atrophy regardless of WMH. In fact, there is increasing evidence that cerebral atherosclerosis is associated with inflammation in the brain which may lead to the deposition of abnormal amyloid and tau proteins^9^.

In this study, we thus evaluated the association between CAC and cortical thickness in a large sample of cognitively normal individuals. We hypothesized that CAC might be correlated with cortical thinning with regional specificity, and their association might be mediated by the presence of WMH.

## Results

### Characteristics of study participants

The mean age of study participants was 64.0 years and 31.5% of participants were female (Table 1). The prevalence of CAC score > 0 was 68.6% (n = 351), including a 36.5% prevalence of CAC scores > 0 to 100 (n = 187), a 19.3% prevalence of CAC scores > 100 to 400 (n = 99), and a 12.7% prevalence of CAC scores of >400 (n = 65).

### Association between CAC scores with cortical thickness

**Figure 1:**
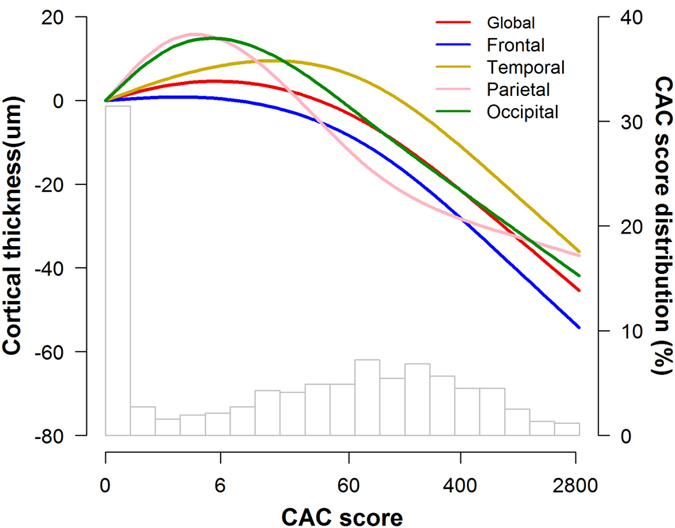
Average difference in cortical thickness in the (**a**) Global, (**b**) Frontal, (**c**) Temporal, (**d**) Parietal and (**e**) Occipital regions by coronary artery calcification scores. The curves were estimated using restricted cubic splines for CAC with knots at the 5^th^, 25^th^, 75^th^ and 95^th^ percentiles of the CAC score sample distributions. The reference value (diamond dot) was set at the 5th percentile (CAC score 0). Models were adjusted for age (continuous), sex, BMI (continuous), education (<high school, ≥high school), smoking status (never, past or current), alcohol intake (never, past or current), hypertension, diabetes, hyperlipidemia, ICV (continuous) and WMH (none or minimal, moderate or severe).

In crude analyses, CAC scores were inversely associated with cortical thickness in the global, frontal, temporal, parietal and occipital regions (Table 2). In multivariable models adjusted for age and sex, BMI, education, smoking status, alcohol intake, hypertension, diabetes, hyperlipidemia, and intracranial volume (ICV), CAC scores were associated with decreased mean cortical thickness in the frontal and occipital regions (Table 2). Adjusting for WMH as a possible mediator did not substantially affect the results. In fully adjusted models, increased CAC scores were associated with decreased cortical thickness globally (*P* for trend = 0.047), in the frontal (*P* for trend = 0.023), and occipital (*P* for trend = 0.048, Table 2). In spline regression models, elevated CAC scores were associated with lower average cortical thickness overall and in all regions, although the association was strongest for the frontal region (Fig. 1).

**Figure 2:**
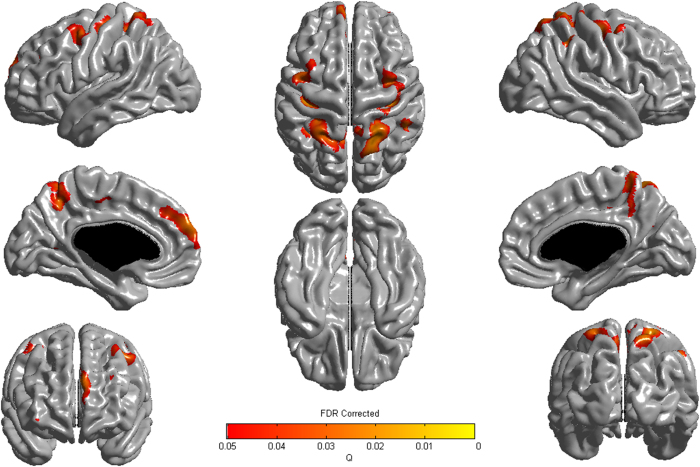
Three-dimensional reconstruction for correlation between coronary artery calcification scores and cortical thickness. The association of higher CAC score with cortical thinning had regional specificity in the bilateral dorsolateral prefrontal, precuneus, superior parietal, and left medial prefrontal regions. The Q-value denotes the FDR-corrected P-value.

Application of the general linear model to vertex-wise cortical thickness showed that elevated CAC scores were associated with cortical thinning in the bilateral dorsolateral prefrontal, precuneus, superior parietal, and left medial prefrontal regions (Fig. 2).

**Figure 3:**
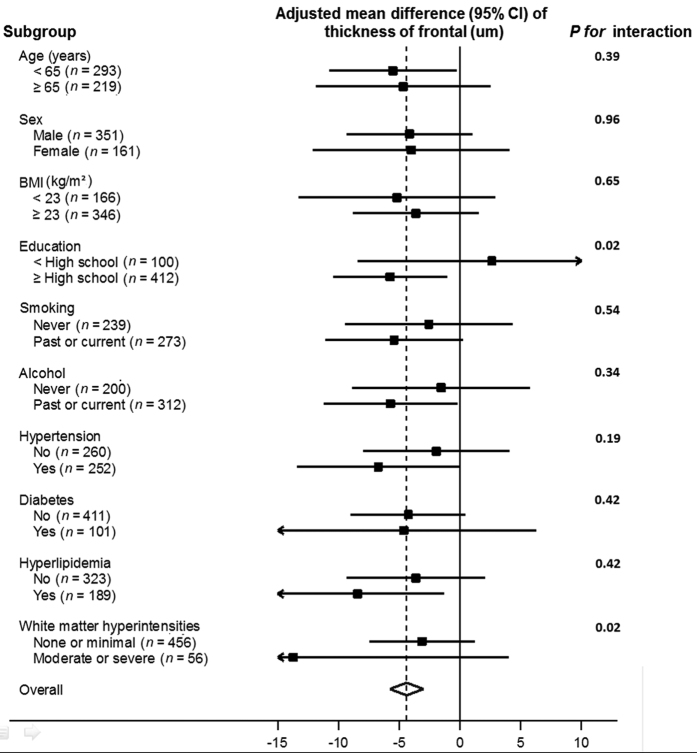
Adjusted average difference in cortical thickness in the frontal region per one unit increase in natural log (CAC score + 1) in clinically relevant subgroups. Models were adjusted for age, sex, BMI, education (<high school, ≥high school), smoking status (never, past or current), alcohol intake (never, past or current), hypertension, diabetes, hyperlipidemia, white matter hyperintensities (none or minimal, moderate or severe) and ICV with CAC exam.

The negative association between CAC and cortical thickness was consistently observed in all subgroups analyzed (Fig. 3) except in participants with ≥ high school education (*P* for interaction = 0.02). The association between CAC scores and cortical thickness was also significantly stronger in participants with moderate or severe WMH compared to those with none or mild WMH (*P* for interaction = 0.02). However, CAC scores were not associated with WMH in both crude and multivariable models (Table 3).

## Discussion

In this study of cognitively normal adults, CAC scores were significantly associated with decreased cortical thickness in the bilateral dorsolateral prefrontal, precuneus, superior parietal, and left medial prefrontal regions, which largely overlap the distribution of default mode network (DMN)^10^. While CAC scores were not associated with WMH severity and the association between CAC scores and cortical thinning remained after adjusting for WMH, the association of CAC with cortical thickness was more prominent in participants with moderate or severe WMH compared to those with none or mild WMH, suggesting that CAC and WMH synergistically affected cortical thinning.

Previous studies showed that CAC scores were associated with lower gray matter (GM) volume, but they suggested that CAC may lead to more diffuse, as opposed to focal, brain damage^2^,^3^,^4^. In our study, the association between CAC scores and cortical thinning showed some regional specificity particularly in key anatomical structures of the DMN. DMN involvement has been implicated in both episodic memory and visual imagery^10^,^11^, and it overlaps with the distribution of amyloid deposition in Alzheimer’s disease (AD)^12^. Indeed, the DMN seems to be affected even in early stages of AD^13^.

Contrary to previous studies^2^,^3^,^4^, we did not find a significant association between CAC scores and cerebral small vessel disease (CSVD) burden measured by WMH severity. This discrepancy may be explained by differences in participant characteristics, with the present study including relatively healthy and cognitively normal participants compared with previous studies. A lack of association between CAC scores and WMH may be related to differences in the pathogenesis and risk factors between small and large artery disease^14^,^15^. In fact, a recent study demonstrated that there is no direct association between large artery disease and WMH^16^. Furthermore, large artery disease is linked to inflammation^17^, while the role of inflammation in small artery disease is controversial^18^.

In our study, the association of CAC scores with cortical thinning remained significant after adjustment for WMH, a finding that suggests that CAC is associated with AD-related cortical thinning rather than CSVD burden. Our finding is consistent with a previous study showing that CAD is associated with cognitive decline independent of WMH^5^. The mechanisms underlying an association between CAC and AD-related cortical thinning remains unknown. CAD risk factors or CAD itself may be associated with increased inflammation in the brain^19^, which may involve glial cell activation and upregulation of inflammatory mediators. Indeed, increased inflammation in the brain has been associated with pathological hallmarks of AD including neuritic plaques and neurofibrillary tangles^9^,^20^. Further studies are needed to understand the mechanisms underlying the association between CAC and cortical thinning.

We also found that the association of CAC with cortical thickness was more prominent in participants with moderate or severe WMH compared to those with none or mild WMH. Since WMH are associated with cortical thinning^8^,^21^, it is possible that WMH may reduce brain reserves making subjects more susceptible to the effects of CAC. Alternatively, CAC may induce AD-related structural abnormalities, which would then have a synergistic effect with WMH. In fact, AD-related structural abnormalities are associated with cerebrovascular disease^22^,^23^, and amyloid and WMH synergistically affect brain atrophy and cognitive function^24^,^25^. Previous studies showed that WMH affected white matter integrity, which in turn leads to secondary axonal and transsynaptic degeneration eventually resulting in cortical thinning^26^. CAC might affect cortical thinning through inflammation and/or AD pathogenesis, and large vessel disease and small vessel disease would synergistically affect brain atrophy via diverse pathways.

The strengths of this study include the use of structural markers of coronary atherosclerosis and of sophisticated measurements of cortical thickness. In addition, the use of high quality clinical measurements and the availability of information on multiple covariates to adjust for established CAD risk factors add to the strength of our findings. However, some limitations should be considered when interpreting these results. First, our study was cross-sectional, precluding claims of causality. Longitudinal studies are needed to determine whether CAC-related brain changes increase over time subsequent to changes in CAC scores. Second, while WMH burden was assessed using structured semequantitative methods, this visual rating scale may not fully reflect the impact of CAC scores on WMH. Third, our study subjects were participants in comprehensive health screening exams not covered by public medical insurance, which might limit the generalizability of this study to the general population.

In conclusion, our findings show that in cognitively normal adults, CAC was associated with cortical thinning in areas related to cognitive function. This association was evident after adjusting for multiple CAD risk factors and for WMH, suggesting that CAC may be more closely related to AD-type disease rather than to CSVD. Follow-up studies are needed to further establish the longitudinal changes in brain structure associated with changes in CAC scores. In addition, it would be worth to examine the association between CAC and cortical thinning among cognitively impaired patients.

## Methods

### Study participants

The study population was comprised of men and women of 40 years of age or older who underwent a comprehensive health screening exam at the Health Promotion Center of the Samsung Medical Center (Seoul, Korea) from January 1, 2009 to December 31, 2013. Since our objective was to evaluate the association between CAC scoring and cortical thickness, the analysis was restricted to subjects who underwent at least one screening exam including both a brain magnetic resonance imaging (MRI) and a coronary CT scan (n = 637). We then excluded participants who had any of the following conditions: missing data on education or mini–mental state examination (MMSE) score (n = 114); scored below the 16^th^ percentile in age-, sex-, and education-matched norms according to the MMSE (n = 24); or had unreliable analyses of cortical thickness due to head motion, blurring of MRI, inadequate registration to a standardized stereotaxic space, misclassification of tissue type, or inexact surface extraction (n = 66). Since study participants could have more than one exclusion criteria, the final sample size was 512 (351 men and 161 women).

### Standard protocol approvals, registrations, and patient consent

This study was approved by the Institutional Review Board at the Samsung Medical Center. The requirement for informed consent was waived because we only used de-identified data collected for clinical purposes during the health screening exams.

### Measurements

Health screening exams were conducted by trained personnel according to a standard protocol. Information regarding this screening program has been described in detail^27^,^28^. At each visit, demographic characteristics, smoking status, alcohol consumption, medical history, and medication use were collected through standardized, self-administered questionnaires. Smoking status was categorized into never, past, or current smoker. Alcohol consumption was categorized into never, past or current. Height and weight were measured by trained nurses with the participants wearing a lightweight hospital gown and no shoes. Body mass index (BMI) was calculated as weight in kilograms divided by height in meters squared. Sitting blood pressure was measured by trained nurses. Hypertension was defined as a systolic blood pressure ≥140 mm Hg, a diastolic blood pressure ≥90 mm Hg, a self-reported history of hypertension, or current use of antihypertensive medications.

Serum total cholesterol, triglycerides, high-density lipoprotein (HDL) cholesterol, and low-density lipoprotein (LDL) cholesterol were determined with an enzymatic colorimetric method. Glucose was measured in fasting blood samples collected after at least 10 hours of fasting. Diabetes mellitus was defined as a fasting serum glucose ≥126 mg/dL, a self-reported history of diabetes, or current use of antidiabetic medications. The Department of Laboratory Medicine and Genetics at Samsung Medical Center has participated in several proficiency testing programs operated by the Korean Association of Quality Assurance for Clinical Laboratory, the Asian Network of Clinical Laboratory Standardization and Harmonization, and the College of American Pathologists.

### Coronary CT scans

Imaging data for CAC scoring was acquired using Brilliance 40 (Philips Medical Systems), VCT LightSpeed 64 (GE Healthcare) or Discovery 750HD (GE Healthcare) multidetector CT scanners. The analysis of the scans was performed on Extended Brilliance Workspace (Philips Medical Systems) or Advantage (GE Healthcare) workstations. CAC scores were calculated as described by Agatston *et al*.^29^.

### Brain MRI scans

All participants underwent a neurological and neuropsychological examination, MMSE testing, and 3D volumetric brain MRI scan. An Achieva 3.0-Tesla MRI scanner (Philips, Best, the Netherlands) was used to acquire 3D T1 Turbo Field Echo (TFE) MRI data using the following imaging parameters: sagittal slice thickness, 1.0 mm with 50% overlap; no gap; repetition time of 9.9 ms; echo time of 4.6 ms; flip angle of 8°; and matrix size of 240 × 240 pixels reconstructed to 480 × 480 over a field view of 240 mm. Radiologists initially inspected all MR images for evidence of brain tumors, lobar infarctions (except lacunar infarctions), and hemorrhages (observed as low intensity areas in T2-weighted images).

T1-weighted MR images were automatically processed using the standard Montreal Neurological Institute image processing software (CIVET) to measure cortical thickness. This software has been validated and extensively described elsewhere^30^,^31^. In summary, native MRI images were first registered into a standardized stereotaxic space using an affine transformation^32^. Non-uniformity artifacts were corrected using the N3 algorithm, and the registered and corrected volumes were classified as white matter (WM), GM, cerebrospinal fluid, and background using an artificial neural net classifier^33^. The surfaces of the inner and outer cortices were automatically extracted by deforming a spherical mesh onto the gray/white boundary of each hemisphere using the Constrained Laplacian-Based Automated Segmentation with Proximities algorithm, which has also been validated and extensively described elsewhere^34^,^35^.

Cortical thickness was calculated as the Euclidean distance between the linked vertices of the inner and outer surfaces after applying an inverse transformation matrix to cortical surfaces and reconstructing them in the native space^34^. To control for brain size, we computed ICV using classified tissue information and a skull mask acquired from the T1-weighted image^36^. ICV was defined as total volume of GM, WM, and cerebrospinal fluid (CSF), with consideration of voxel dimension. Classified GM, WM, CSF, and background within the mask were transformed back into individual native space.

To compare the thicknesses of corresponding regions among participants, the thicknesses were spatially registered on an unbiased iterative group template by matching the sulcal folding pattern using surface-based registration involving sphere-to-sphere warping^37^. For global and lobar regional analyses, we used the lobe-parcellated group template that had been previously divided into frontal, temporal, parietal, and occipital lobes using SUMA (http://afni.nimh.nih.gov)^35^. Average values of thickness of the whole vertex in each hemisphere and lobar region were used for global analysis.

### WMH visual rating scale

We used a modified Fezekas scale for visual rating of WMH^38^. On this scale, periventricular WMH (PWMH) were classified as P1 (cap or band < 5 mm), P2 (5 mm ≤ cap or band < 10 mm), and P3 (cap or band ≥ 10 mm); deep WMH (DWMH) were classified into D1 (maximum diameter of deep white matter lesion < 10 mm), D2 (10 mm ≤ lesion < 25 mm), and D3 (≥25mm). The intra-class correlation coefficients for inter-rater reliability of the WMH visual rating scale ranged from 0.73 and 0.91^39^. The WMH visual rating scale also correlated well with automated measured WMH volume^40^. WMH ratings were combined to give a final classification of minimal (combinations of D1 with P1 [D1P1] and D1 with P2 [D1P2]) moderate (D2P1, D3P1, D2P2, D3P2, D1P3, and D2P3) or severe (D3P3). This classification discriminates the presence of vascular risk factors and the severity of cerebrovascular disease markers^40^.

### Statistical analysis

Linear mixed models that allowed for random variability between both hemispheres of the same participant were used to evaluate the association between CAC scores and cortical thickness. For the main analyses, we calculated the mean difference and 95% confidence interval (CI) in cortical thickness (global, frontal, temporal, parietal, and occipital regions) associated with CAC score increases across participants. Since CAC scores are markedly right skewed, we used log_e_-transformed (CAC score + 1) as the exposure. As secondary analyses, we divided CAC scores into four categories (0, >0–100, >100–400 and >400)^41^. In addition, we modeled CAC scores as a continuous variable using restricted cubic splines with knots at the 5^th^, 35^th^, 65^th^ and 95^th^ percentiles of the sample distributions to provide a flexible estimate of the dose-response relationship between CAC score and cortical thickness. As a secondary analysis, we used logistic regression to calculate the odds ratios (with 95% CI) for moderate or severe WMH associated with different categories of CAC.

For multivariable models, we adjusted for potential confounding factors including age (continuous), sex, BMI (continuous), education (<high school, ≥high school), smoking status (never, past, current), alcohol intake (never, past or current), hypertension, diabetes, hyperlipidemia, ICV (continuous), and WMH (none or minimal, moderate or severe).

In addition, we explored the association of CAC with cortical thickness in pre-specified subgroups defined by age (<65 vs. ≥65 years), sex (men vs. women), BMI, education (<high school vs. ≥high school), smoking status (never vs. past or current smokers), alcohol intake (never vs. past or current drinkers), hypertension, diabetes, hyperlipidemia, ICV, and WMH (none or minimal vs. moderate or severe). All reported P-values were two-sided and the significance level was set at 0.05. Statistical analyses were performed using STATA version 13 (StataCorp LP, College Station, TX, USA).

For cortical thickness analyses, we used a MATLAB-based toolbox (available at the University of Chicago website: http://galton.uchicago.edu/faculty/InMemoriam/worsley/research/surfstat/). Diffusion smoothing with a full-width half-maximum of 20 mm was used to blur each cortical thickness map, leading to increased signal-to-noise ratio and statistical power^30^. In order to analyze the localized differences and the statistical map of cortical thickness on the surface model, linear mixed models were performed vertex-by-vertex after controlling for age, sex, BMI, education, smoking status, alcohol intake, hypertension, diabetes, hyperlipidemia, ICV, and WMH. The resulting statistical maps were thresholded using a false discovery rate (FDR)^42^ with a Q-value of 0.05 after pooling the P-values from regression analyses.

## Additional Information

**How to cite this article**: Lee, J. S. *et al*. Coronary artery calcium is associated with cortical thinning in cognitively normal individuals. *Sci. Rep.* **6**, 34722; doi: 10.1038/srep34722 (2016).

## Figures and Tables

**Table 1:** Characteristics of study participants (n = 512).

Characteristic	N (%) or Mean (SD)
Age (years)	64.0 (6.9)
Female sex	161 (31.5)
BMI (kg/m^2^)	24.2 (2.7)
Total cholesterol (mg/dL)	189.4 (38.3)
HDL cholesterol (mg/dL)	54.8 (14.9)
Triglycerides (mg/dL)	115.3 (64.8)
Smoking	
Never	239 (46.7)
Past or current	273 (53.3)
Alcohol	
Never	200 (39.1)
Past or current	312 (60.9)
Education	
<High school	100 (19.5)
≥High school	412 (80.5)
K-MMSE score	28.2 (1.6)
Systolic blood pressure (mmHg)	122.7 (16.9)
Diastolic blood pressure (mmHg)	74.8 (10.1)
Hypertension	252 (49.2)
Diabetes	101 (19.7)
Hyperlipidemia	189 (36.9)
CAC score > 0	351 (68.6)
ICV (cm^3^)	1.4 (0.1)
WMH	
None	230 (44.9)
Mild	226 (44.1)
Moderate	40 (7.8)
Severe	16 (3.1)
Left cortical thickness (mm)	
Hemispheric	3.0 (0.1)
Frontal	3.1 (0.1)
Temporal	3.2 (0.2)
Parietal	2.9 (0.1)
Occipital	2.7 (0.1)
Right cortical thickness (mm)	
Hemispheric	3.0 (0.1)
Frontal	3.1 (0.1)
Temporal	3.2 (0.1)
Parietal	2.9 (0.1)
Occipital	2.7 (0.1)

Abbreviations: BMI = body mass index; HDL = high-density lipoprotein; K-MMSE = Korean mini-mental state examination; CAC = coronary artery calcium; ICV = intracranial volume; WMH = white matter hyperintensities.

**Table 2:** Average difference (95% CIs) in cortical thickness (μm) associated with coronary artery calcification scores* (n = 512).

	Global	Frontal	Temporal	Parietal	Occipital
Crude model					
Continuous CAC score	−8.6 (−12.3, −4.9)	−10.2 (−10.1, −6.2)	−7.8 (−13.1, −2.5)	−9.2 (−13.8, −4.5)	−6.1 (−10.5, −1.8)
CAC score category					
0	*reference*	*reference*	*reference*	*reference*	*reference*
>0–100	−13.8 (−36.2, 8.6)	−17.3 (−41.5, 6.9)	−16.1 (−48.2, 16.0)	−10.1 (−38.3, 18.0)	0.2 (−25.8, 26.3)
>100–400	−42.1 (−68.8, −15.5)	−53.5 (−82.3, −24.7)	−25.1 (−63.2, 13.1)	−50.7 (−84.2, −17.3)	−26.9 (−58.0, 4.1)
>400	−67.2 (−97.8, −36.5)	−76.2 (−109.2, −43.1)	−65.1 (−108.9, −21.2)	−69.7 (−108.1, −31.1)	−52.1 (−87.8, −16.4)
*P* for trend	<0.001	<0.001	0.005	<0.001	0.002
Model 1					
Continuous CAC score	−3.6 (−7.8, 0.6)	−4.7 (−9.3, −0.1)	−1.9 (−8.1, 4.4)	−4.2 (−9.6, 1.2)	−3.9 (−8.7, 0.9)
CAC score category					
0	*reference*	*reference*	*reference*	*reference*	*reference*
>0–100	4.1 (−18.4, 26.5)	1.6 (−22.9, 26.1)	4.8 (−28.6, 38.2)	8.6 (−20.3, 37.4)	11.2 (−14.5, 36.8)
>100–400	−14.0 (−42.1, 14.1)	−22.6 (−53.2, 8.1)	8.2 (−33.6, 50.1)	−21.7 (−57.8, 14.4)	−13.9 (−46.0, 18.2)
>400	−30.1 (−63.1, 2.8)	−35.7 (−71.7, 0.3)	−19.5 (−68.6, 29.6)	−34.5 (−76.9, 7.9)	−34.1 (−72.9, 3.6)
*P* for trend	0.052	0.026	0.604	0.055	0.049
Model 2					
Continuous CAC score	−3.6 (−7.8, 0.5)	−4.8 (−9.3, −0.2)	−1.9 (−8.1, 4.2)	−4.2 (−9.6, 1.2)	−3.9 (−8.7, 0.9)
CAC score category					
0	*reference*	*reference*	*reference*	*reference*	*reference*
>0–100	4.3 (−17.9, 26.7)	2.1 (−22.3, 26.4)	5.3 (−27.9, 38.5)	8.6 (−20.3, 37.4)	11.3 (−14.3, 36.9)
>100–400	−13.8 (−41.7, 14.1)	−22.3 (−52.8, 8.1)	8.5 (−33.0, 50.1)	−21.7 (−57.8, 14.4)	−13.8 (−45.9, 18.3)
>400	−30.7 (−63.5, 2.1)	−36.4 (−72.2, −0.7)	−20.5 (−69.3, 28.3)	−34.5 (−76.9, 7.8)	−34.4 (−72.1, 3.3)
*P* for trend	0.047	0.023	0.580	0.055	0.048

Model 1: Adjusted for age, sex, BMI, education, smoking status, alcohol intake, hypertension, diabetes, hyperlipidemia, and ICV. Model 2: Further adjusted for WMH category. Abbreviations: CAC = coronary artery calcium; BMI = body mass index; ICV = intracranial volume; WMH = white matter hyperintensities.

^*^Adjusted mean difference and 95% confidence intervals (CIs) were obtained from linear mixed models using natural log (CAC score + 1) as the exposure.

**Table 3:** Odds ratios (95% CIs) for moderate or severe degree of white matter hyperintensities associated with CAC scores.

	OR (95% CI)
Crude model	
Continuous CAC score	1.09 (0.98, 1.22)
CAC score category	
0	*reference*
>0–100	1.65 (0.79, 3.46)
>100–400	1.88 (0.82, 4.30)
>400	2.00 (0.80, 4.99)
*P* for trend	0.103
Adjusted† model	
Continuous CAC score	1.00 (0.87, 1.15)
CAC score category	
0	*reference*
>0–100	1.22 (0.54, 2.77)
>100–400	1.22 (0.46, 3.19)
>400	0.99 (0.32, 2.99)
*P* for trend	0.980

^†^Adjusted for age and sex, BMI, education, smoking status, alcohol intake, hypertension, diabetes, hyperlipidemia, and ICV.

Abbreviations: CAC = coronary artery calcium; BMI = body mass index; ICV = intracranial volume.
